# Supplementary material for: Durable and self-hydrating tungsten carbide-based composite polymer electrolyte membrane fuel cells
Source: Nat Commun. 2017 Sep 4;8:418. doi: 10.1038/s41467-017-00507-6 (PMC5583391; doi:10.1038/s41467-017-00507-6)
Supplement: Supplementary file 1 — Supplementary Information [file 41467_2017_507_MOESM1_ESM.pdf]

Description of Supplementary Files

File Name: Supplementary Information
Description: Supplemental Figures, Supplementary Notes, Supplementary Tables and Supplementary References

File Name: Peer Review File

Supplementary Note 1. Synthesis of early transition metal carbides nanoparticles supported on carbon spheres

The conventional synthesis of carbides or nitrides entails normally through high-temperature carburization or nitridation of the metals, leading to low surface area. One of the most facile routes to high-surface-area transition metal carbides is attributed to Lee et al.,[1] who developed a temperature-programmed reduction-carburization (TPRC) method to form carbides from precursor oxides under a wide range of conditions. Unfortunately, the unstable mesoporous structure limits the application of this material, especially for high temperature and high pressure reactions.

Recently, an old technique[2] has gained renewed interest, whereby biomass is hydrothermally treated in water under relatively mild conditions providing bulk, mesoporous, or nanostructured carbon materials.[3,4] Cui et al. found that the presence of metal ions effectively accelerates the hydrothermal carbonization (HTC) of starch with shorter reaction times and control of particle shape of carbon materials.[5] Sun and Li applied hydrothermal reduction to encapsulate noble metal nanoparticles into the core of carbon spheres.[6]

Inspired by these works, we believe that the formation of solid carbon by HTC could lead to carbide or nitride nanoparticles through the reduction-carburization or nitridation processes, respectively. Here we present a preparation method for production of nano carbides particles dispersed on carbon materials. The synthetic strategy is shown in **Figure 1**, and all experimental details are summarized in the methods section.

Supplementary Figure 1 shows a top view of the as-prepared nano-WC sample, which has a smooth spherical structure with a diameter of 3-5 μm containing well-dispersed WC nanoparticles on its surface. We used focused ion beam to mill a selected region to reveal the cross-sectional

morphology of individual carbon spheres. Although W signal is found across the entire sphere by EDX mapping, nanoparticles of WC are only observed on the surface of carbon spheres. Due to the low resolution of the SEM technique, bright spots on the carbon sphere surface are not necessarily individual particles of WC, since several nanoparticles closely packed on a support surface may also result in a bright spot in SEM images at low magnification. We have further carried out TEM/STEM analysis to characterize the dispersion of WC on carbon spheres, as shown in **Figure 1b,c,d** and **Supplementary Figure 2**.

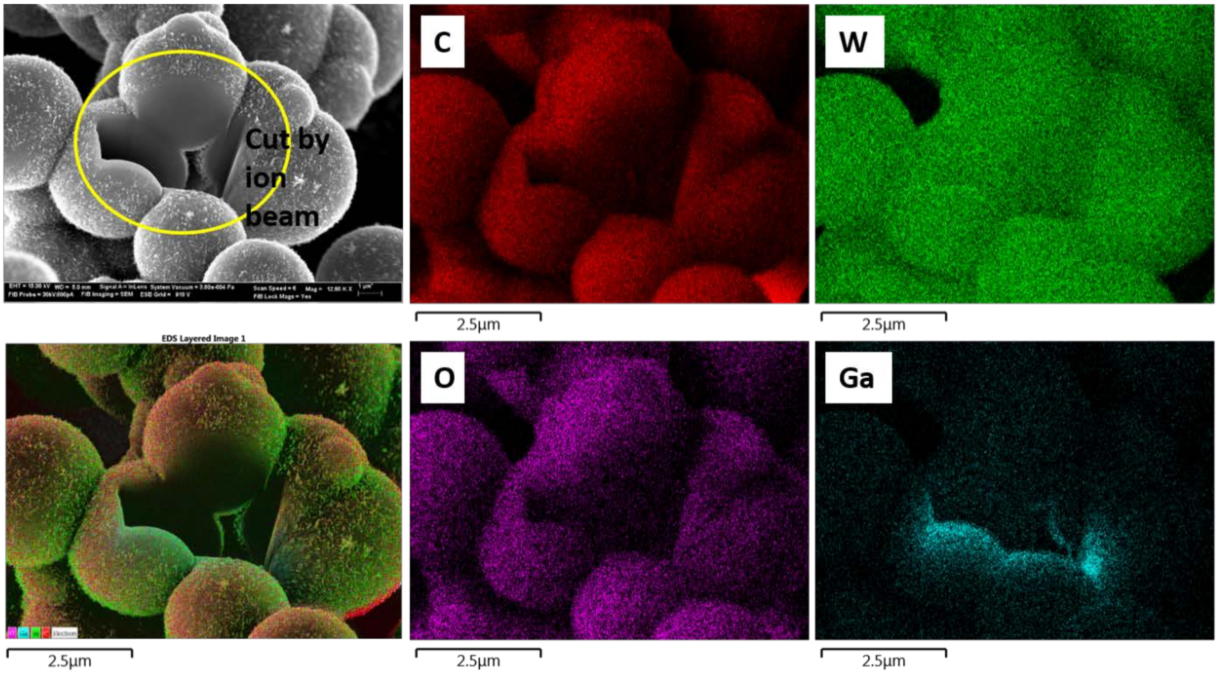

Supplementary Figure 1. SEM image and EDX mapping of WC nanoparticles supported on carbon spheres cut by a focused ion beam. The sample was milled using a Ga+ ion beam with a selected region (7μm × 7μm × 5μm, lenth × width × depth) operated with an energy of 30 kV and a current of 600 pA.

Both HAADF-STEM and TEM investigations confirm that the WC nanoparticles are uniformly dispersed on the carbon sphere with a narrow size distribution. We have also conducted STEM tomography analysis of the as-prepared nano-WC sample. STEM images were recorded by tilting

the sample from -65° to +55° with 1° increment. Representative 3D rendering of the reconstructed image is shown in **Figure 2c**, which again shows homogeneously dispersed WC nanoparticles.

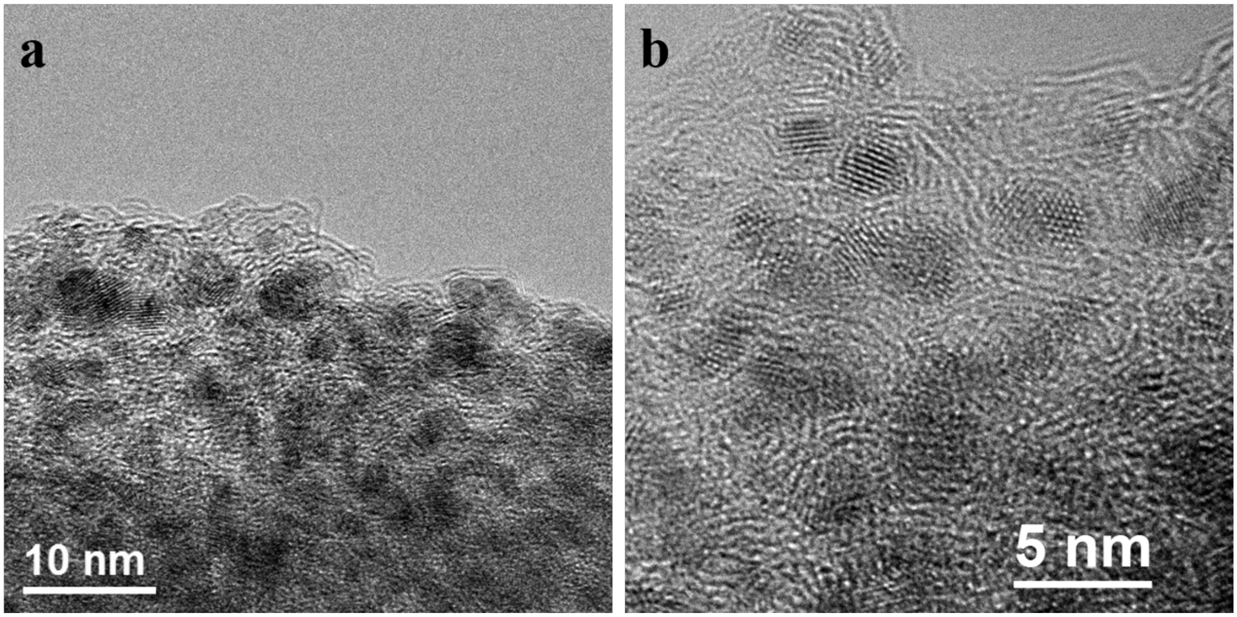

Supplementary Figure 2. Bright field TEM images of nano-WC located on a carbon sphere.

In order to confirm the crystalline structure of our as-prepared nano-WC nanoparticles, we have applied powder XRD analysis together with lattice indexing of HRTEM, as shown in **Supplementary Figure 3** and **Figure 2C**.

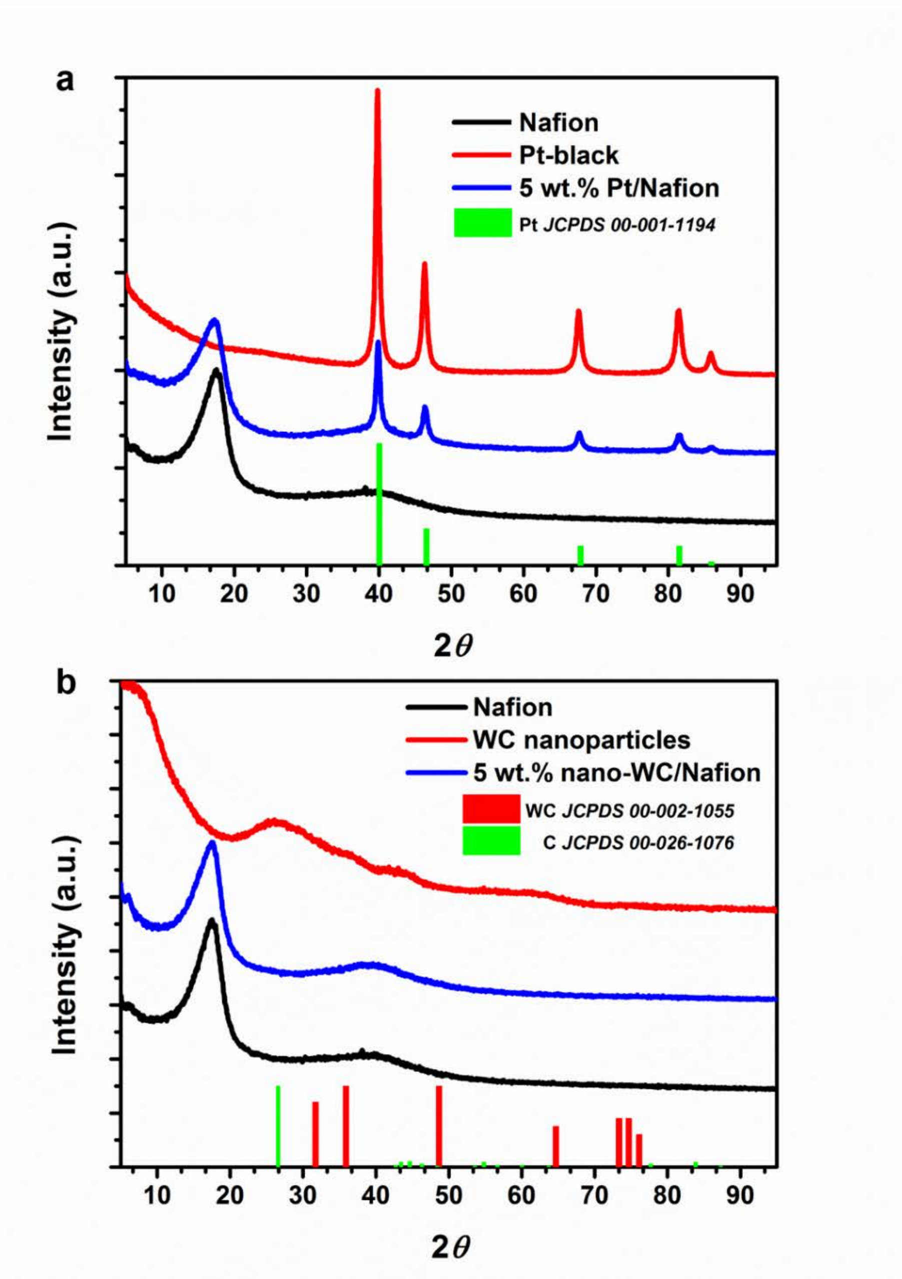

Supplementary Figure 3. Powder X-ray diffraction characterization of various materials indicated in the legend. **a**, XRD patterns of Pt-black, composite membrane containing 5wt.% Pt black compared to recast Nafion. Pt patterns were assigned according to the Joint Committee on Powder Diffraction Standards (JCPDS) 00-001-1194 and **b**, XRD patterns of WC nanoparticles, composite membrane containing 5 wt.% WC nanoparticles compared with recast Nafion membrane. WC and carbon patterns were assigned according to the JCPDS files of 00-002-1055 and 00-026-1076, respectively.

The surface properties of nano-WC catalyst were further analyzed by X-ray photoelectron spectroscopy (XPS). **Supplementary Figure 4** summarizes the XPS spectra of the W4*f*, C1*s*, O1*s* core level and fermi level of commercial WO_3 (comm-WO_3), commercial WC (comm-WC), nano-WO_x (sample collected after the HTC step was annealed in He at 700 °C) and nano-WC (passivated in 5% O_2-He before transferred to XPS analysis). It is clear that the surface of nano-WC prepared by the two-step synthetic protocol is dominated by WC: W4*f* spectrum of nano-WC shows the typical carbidic bonding at about 31.6 and 33.7 eV which are the doublets of $4f_{7/2}$ and $4f_{5/2}$ electrons, consistent with the range values reported in the literature for the tungsten carbide surfaces;[7] the C1*s* spectrum of nano-WC shows a minor shoulder peak at around 282.6 eV which is also found in comm-WC sample. It is in agreement with the values assigned to carbidic carbon peak in literature; the density of electronic states of WC nanoparticles (nano-WC) is close to that of the commercial WC exhibiting a metallic nature with high density at the Fermi level (**Supplementary Figure 4d**); the O1*s* spectrum of nano-WC shows very minor oxygen features, indicating a tungsten carbide dominated surface.

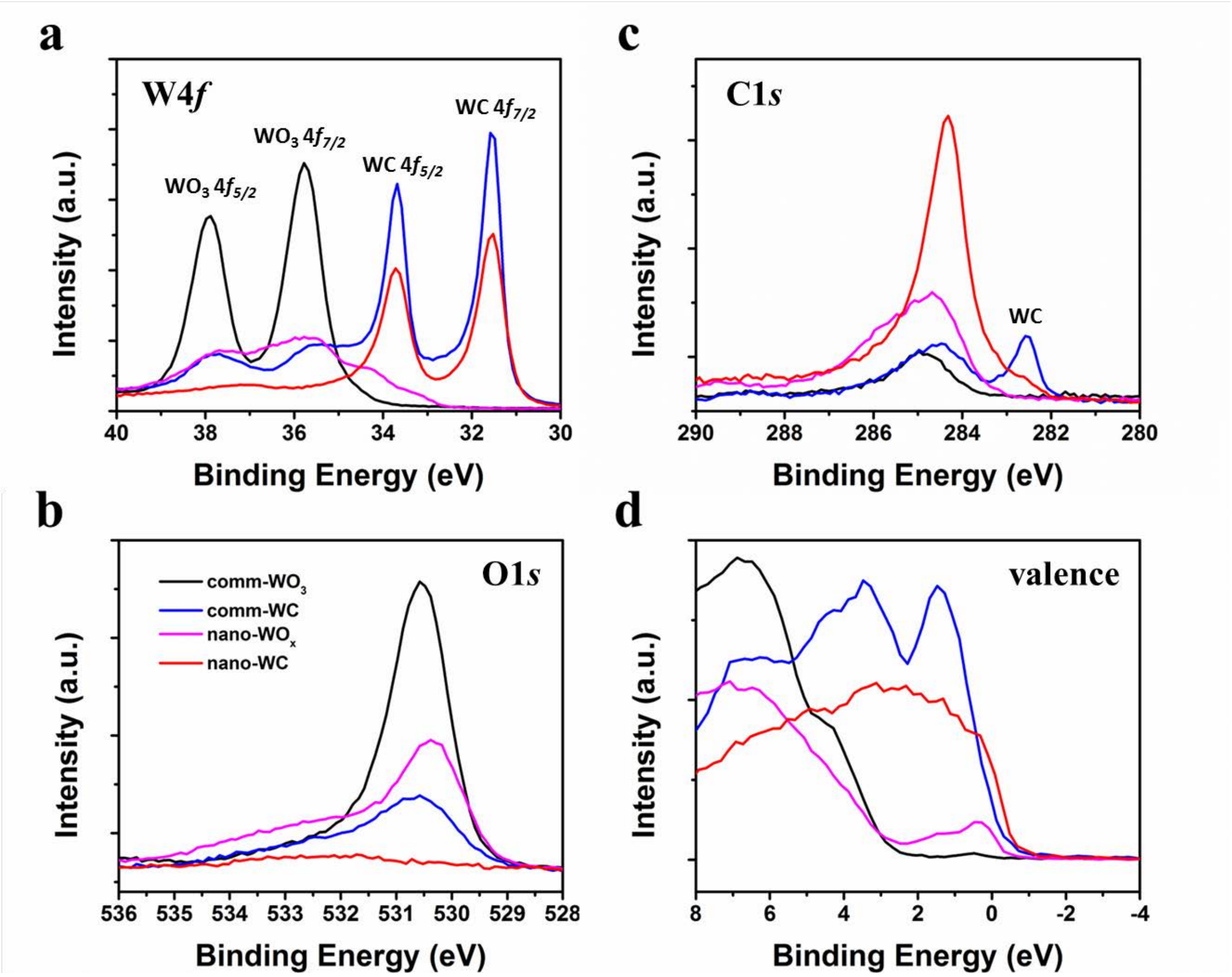

Supplementary Figure 4. W4f, O1s, C1s core-level and valence spectra of comm-WO_3, comm-WC, nano-WO_x and nano-WC.

Ganesan and Lee reported a method of W_2C preparation by heating a mixture of a resorcinol-formaldehyde polymer and ammonium metatungstate.[8] Yan and Shen also reported a similar method by heating a mixture of ion-exchange resin and W precursor.[9] We believe that "graphitic coke" differs from our materials, because the WC nanoparticles of prior work are not catalytically active in their applications (ORR and methanol oxidation). We have also annealed the samples collected after the HTC step in inert gas (He) at different temperatures (**Supplementary Figure**

5). Apparently, none of the patterns match well those of α-WC but are similar to diffraction patterns of tetragonal WO_3 structure. This indicates that the reduction carburization is a facile route to carburize the W precursor into the interstitial structure.

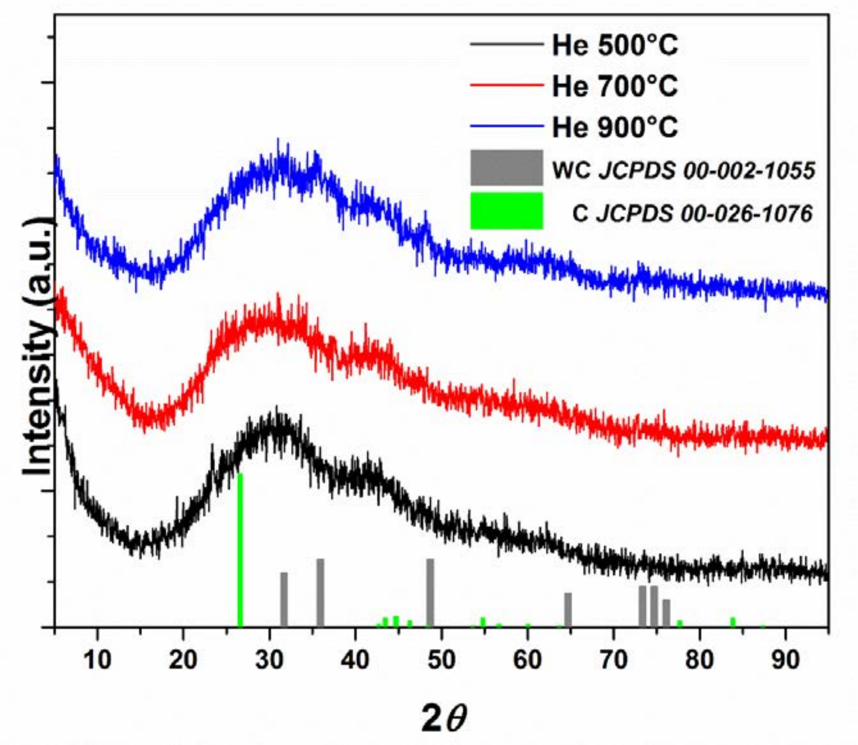

Supplementary Figure 5. Powder X-ray diffraction characterization of various materials indicated in the legend. Sample collected after the HTC step was further annealed in flowing He gas at 500, 700 and 900 °C. Standard diffraction patterns of WC (JCPDS 00-002-1055) and C (JCPDS 00-026-1076) are included as references.

We have also conducted thermogravimetric analysis (TGA) of the nano-WC sample under flowing air (see **Supplementary Figure 6**). With the assumption that all W is oxidized to WO_3 and the carbon sphere is combusted, we estimate the total loading of W in nano-WC to be around 60 wt.%. We have further calculated the surface elemental concentration of nano-WC from the XPS survey spectrum, which can detect 2-5 nm in depth from the surface (**Supplementary Table**

1). The nano-WC contains about 52 wt.% of W on top atomic layers of WC and carbon sphere. We estimate that about 86% of W in nano-WC is carburized near the surface during synthesis.

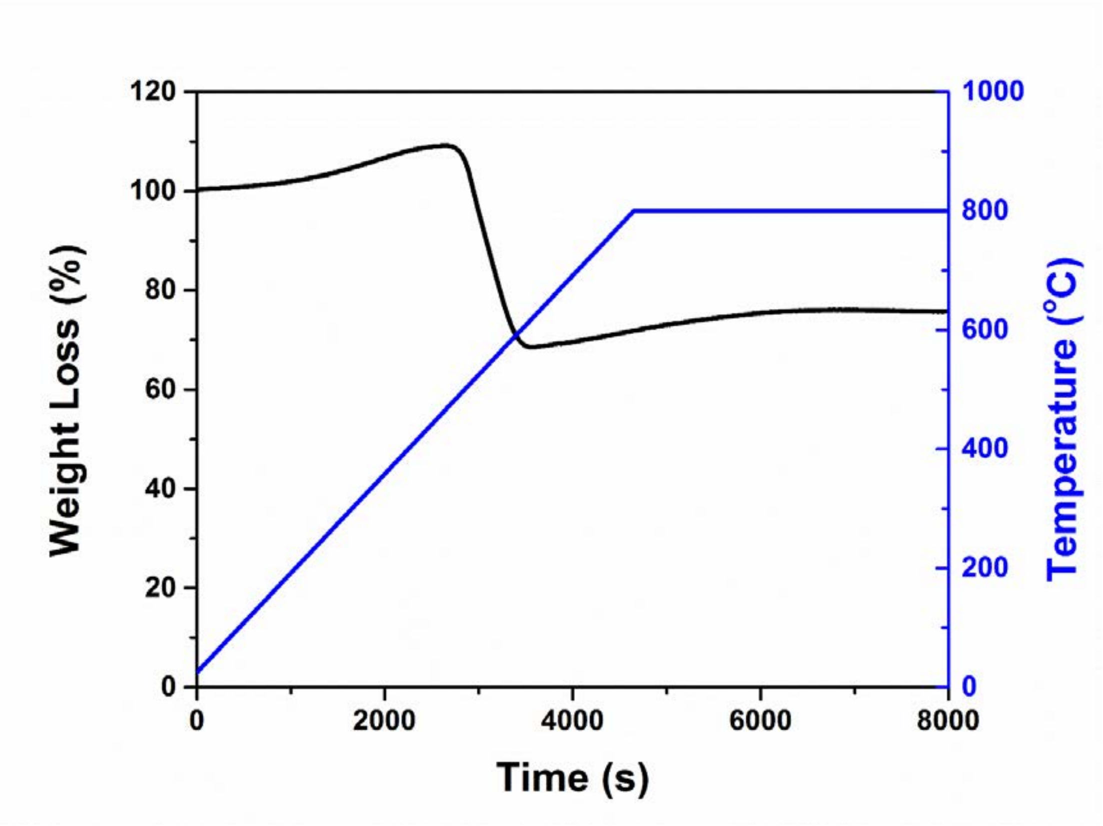

Supplementary Figure 6. Thermogravimetric analysis result of a nano-WC sample.

Supplementary Table 1. Surface atomic concentration of elements of nano-WC and nano-WOx from XPS survey spectra.

Catalyst	Atomic percentage (%)		
	C	O	W
nano-WC	91.09	2.22	6.69
nano-WO_x	63.79	27.59	8.62

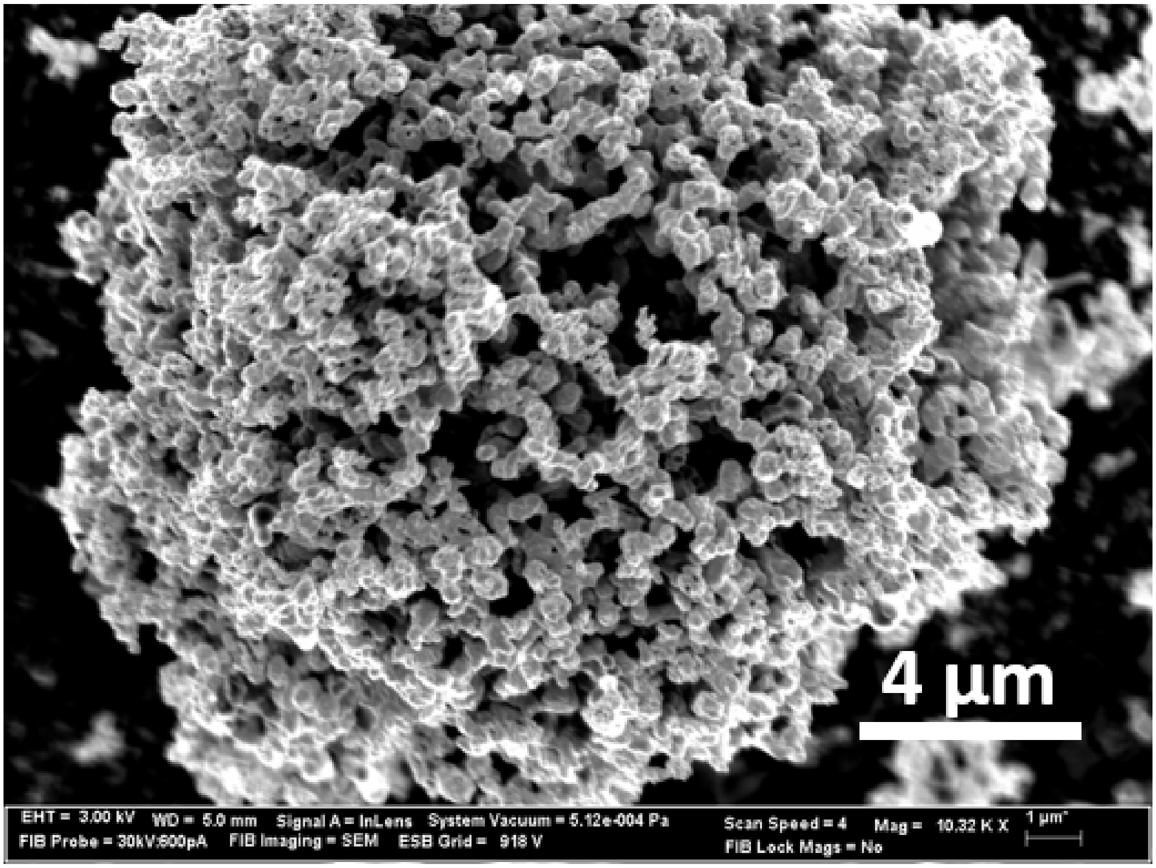

Supplementary Figure 7. SEM image of commercial WC catalyst.

To demonstrate the generality of our approach, we present here images of different TMC nanoparticles prepared by our two-step synthetic method. **Supplementary Figure 8** shows representative SEM and TEM images of nano Mo_2C particles. As shown in **Supplementary Figure 8b**, which is a fragment of as prepared carbon sphere grounded and deposited on the grid for TEM analysis, the black dots dispersed on carbon sphere are assigned to Mo_2C. This was confirmed by TEM analysis at higher magnification (**Supplementary Figure 8c**) and atomic resolution (**Supplementary Figure 8d**) with lattice indexing, which shows the hexagonal closed packed *ß*-Mo_2C structure.

Considering the well-known similarities of carburization, nitridation and sulfidation,[10] it is possible that by changing gas precursors in the second step of our approach, nanoparticles of nitrides and sulfides can also be formed. Further work will focus on such materials.

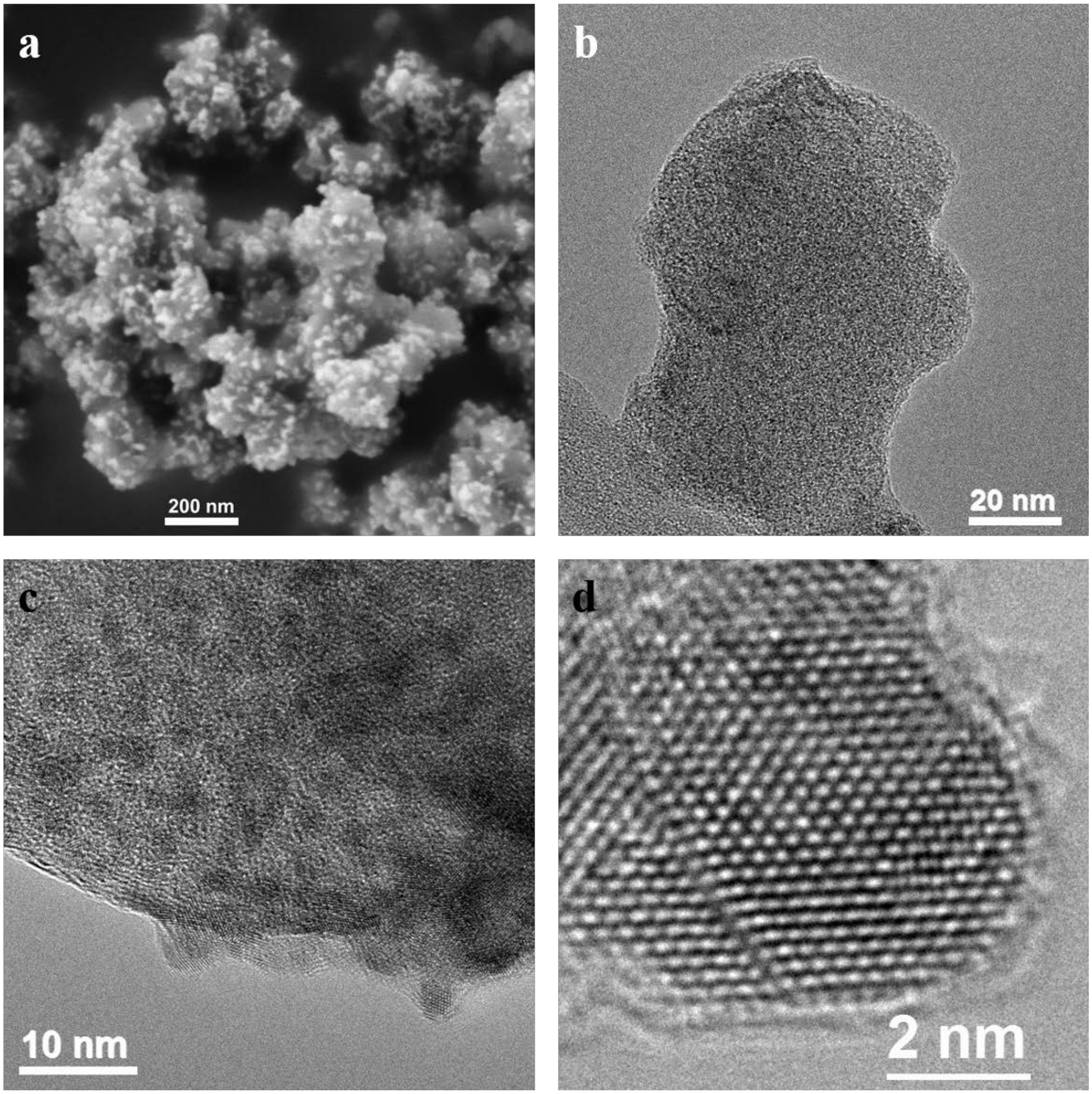

Supplementary Figure 8. Electron microscopy analysis of Mo_2C nanoparticles prepared by HTC-TPRC method. a, SEM image of Mo_2C nanoparticles (bright dots) dispersed on carbon (gray spheres); b, low magnification TEM image of a representative carbon sphere fragment with highly dispersed Mo_2C nanoparticles (black dots); c, high magnification TEM image observing the edge region of the carbon sphere with narrow distributed Mo_2C nanoparticles (black spots); d, high resolution TEM image of a representative Mo_2C nanoparticle.

Supplementary Note 2. Recast composite Nafion membrane for proton exchange membrane fuel cell

Preparation of recast composite Nafion membranes. An inorganic/organic composite self-hydrating polymer electrolyte membrane was developed by Watanabe et al.[11] In that work, the nanometer-size Pt is dispersed into the Nafion resin to catalyze the oxidation of crossover hydrogen with oxygen and generate water to hydrate the membrane. Because of the high cost of Pt and its negative effect to the durability of membrane (see paper), we developed a nano-WC catalyst for self-hydration as a replacement to Pt. 20 ml of 5% Nafion solution (D-521, ≥0.92 meq/g, Alfa Aesar) was dried at 60 °C to vaporize the solvent. The Nafion® resin was then dissolved in dimethylacetamide (DMAC) to form Nafion/DMAC solution and 47 mg of the previously prepared WC NPs was added to it. The mixture was sonicated for at least 2 hours to mix with Nafion. Then the nano-WC/Nafion solution was poured onto a glass plate and heated in an air oven at 120 °C for 4 hours, and in a vacuum oven at 150 °C for 2 hours. The cured membrane of thickness 50 μm was then lifted off the glass plate and immersed in 0.5 M sulfuric acid for 2 hours, and rinsed with DI water. Composite membranes with Pt black, commercial WC and recast Nafion were prepared using the same procedure.

Fuel cell performance tests. The composite membranes were hot-pressed between gas diffusion electrodes (GDEs) with 0.3 mg/cm^2 Pt loading at 130 °C for 2 mins to fabricate the membrane electrode assembly (MEA). The MEA's performance was then tested in a 5 cm^2 fuel cell. All tests were conducted at a cell temperature of 70 °C. H_2 and O_2 flow rates were 200 mL/min and 400 mL/min, respectively. The gas supply lines were maintained at 75 ^oC to prevent condensation of water vapor. The temperature of humidifiers was controlled at 70, 55, 41 and 14 ^oC (cooling water

temperature) to achieve relative humidity (RH) of 100, 50, 25 and 5%, respectively. The RH was calculated from

$$\mathrm{RH} = P_{H2O}/P^{*}_{H2O}$$

Here P_{H2O} is the ratio of the partial pressure of water vapor in the mixture to the equilibrium vapor pressure of water (P^{*}_{H2O}) at a given temperature.

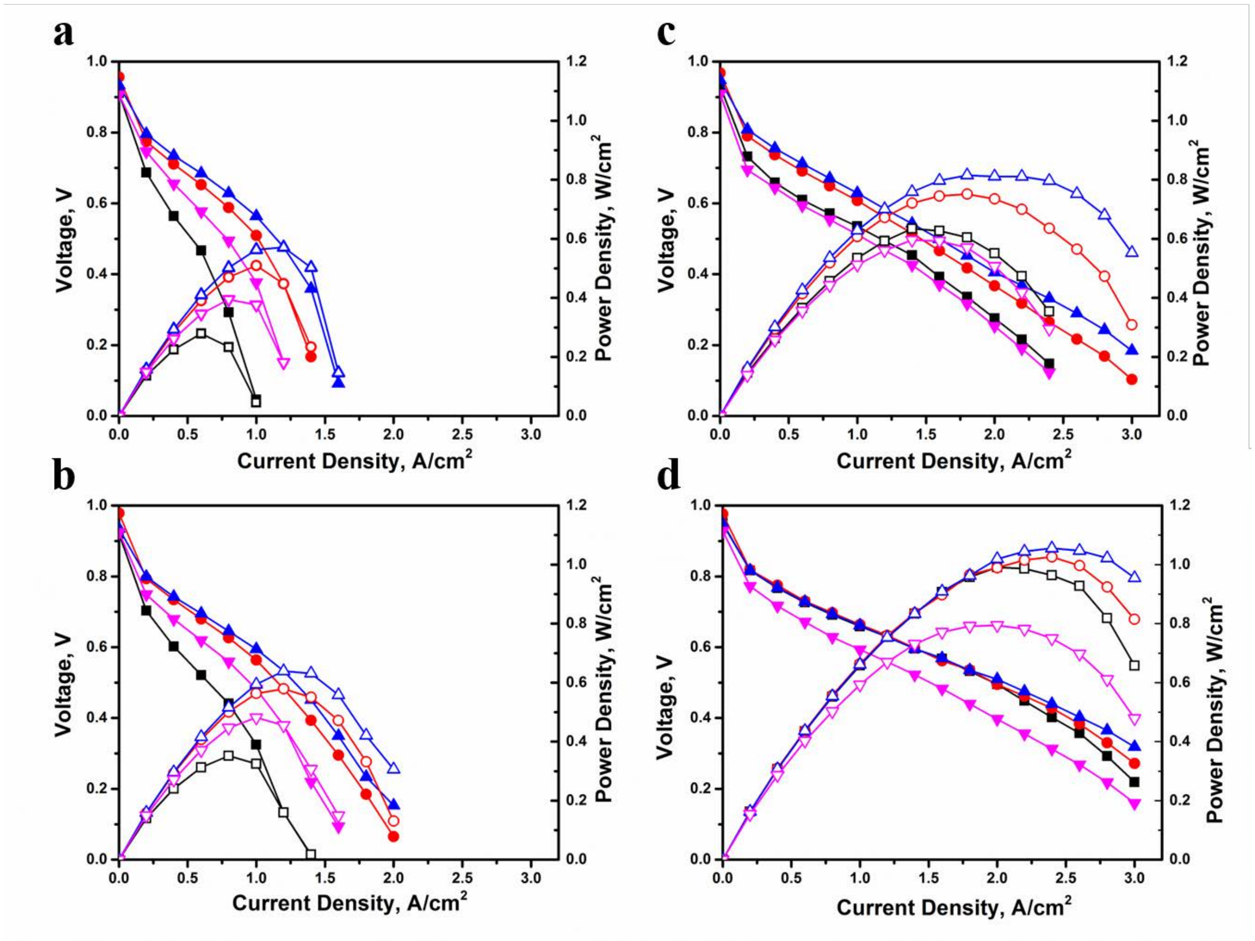

Supplementary Figure 9. Initial fuel cell performance consisting of baseline recast Nafion membrane (black) and composite membranes incorporating nano-WC (red), commercial WC (pink), and Pt black catalysts (blue). The polarization I-V evaluation of the fuel cell was conducted and controlled by a fuel cell test station from Arbin Instruments. The H_2 and O_2 humidifiers were maintained at 70, 55, 41, and 14 °C while the fuel cell temperature was set to 70 °C such that the relative humidity of the inlet gases was 100, 50, 25, and 5%. Gas supply lines temperature were maintained 5 °C higher than the fuel cell temperature to prevent condensation of water vapor. Hydrogen fuel and oxygen were fed in co-flow to the fuel cell. H_2 and O_2 flow rates were 200 ml/min and 400 ml/min, respectively.

Supplementary Figure 9 shows the fuel cell performance of recast Nafion, Pt Nafion, nano-WC/Nafion and commercial WC/Nafion membranes. The Pt/Nafion membrane (blue) shows the least decrease in performance when the humidity drops from 100% RH to 5% RH. Our nano-WC/Nafion shows similar improvement as the Pt/Nafion but less than Pt black due to the lower activity of nano-WC catalyst compared to that of Pt black. However, the improvement is still significant considering the low cost of nano-WC catalyst and the positive effect on membrane durability. Recast Nafion without self-hydrating function shows the largest decrease in performance (from 1 W/cm^2 at 100% RH to 0.3 W/cm^2 at 5% RH).

The degradation rate for regions i-iii in **Supplementary Figure 10c** is 1.38±0.01 mV/h, 6.09±0.04 mV/h and 14.3±0.14 mV/h, respectively (**Supplementary Table 2**). This degradation is due to major defects formed during the test from higher gas crossover. The accelerated durability tests were conducted according to the DOE protocol at 90 °C and 35% RH.[12] Fuel cells were first conditioned at 1A/cm^2 for 8 hours at 100% RH and 70 °C. Then the fuel cell temperature was raised to 90 °C, and the relative humidity was reduced to 35%. When the fuel cell and humidifiers reached the desired temperature, the fuel cell was switched to OCV, and the durability test started. The OCV was recorded for evaluation of durability. This test is designed to be much faster than the conventional one so that the lifespan of different membranes can be studied in laboratories, usually within 100 to 300 hours. Since the failure of Pt/Nafion membrane happens within 100 hours, we conducted tests for 100 hours.

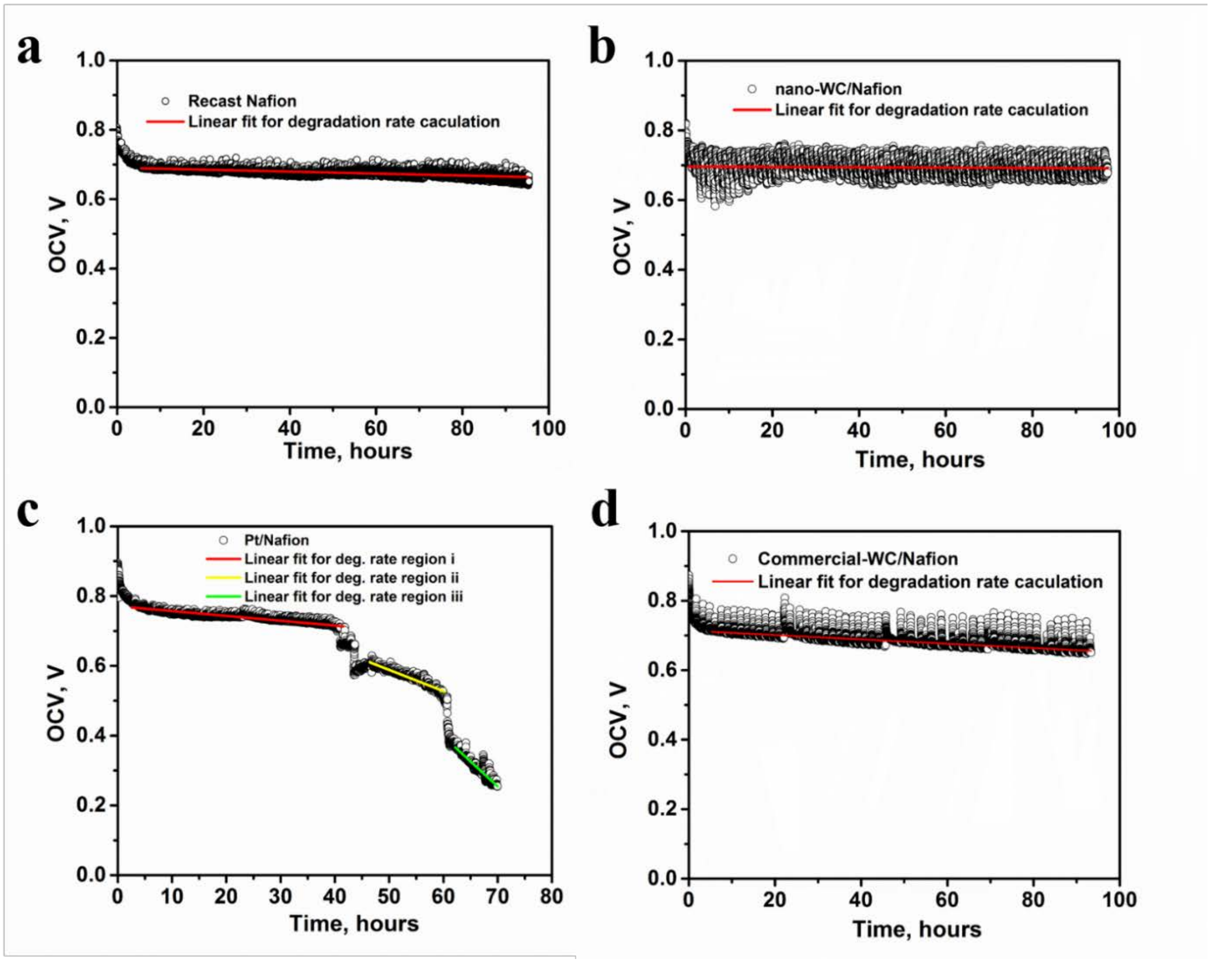

Supplementary Figure 10. Linear fit of accelerated durability tests of **a,** recast Nafion membrane; **b,** composite membranes incorporating nano-WC catalyst; **c,** composite membranes incorporating Pt catalyst; and **d**, composite membranes incorporating commercial WC catalyst.

Supplementary Table 2. Linear fit of accelerated durability tests.

Fit	Nafion	nano-WC	comm. WC	Pt particles		
				region i	region ii	region iii
Slope (mV/h)	-0.28±0.003	-0.05±0.008	-0.62±0.005	-1.38±0.01	-6.1±0.04	-14.3±0.14
Residual Sum of Squares	0.63344	7.45837	1.55244	0.3086	0.07921	0.0818
R-Square	0.47382	0.0031	0.6309	0.7867	0.9250	0.9222

The failure of Pt/Nafion is repeatable based on our tests on multiple samples (**Supplementary Figure 11**). All three samples showed similar trends and failed at ~70 hours. The slight variability in the OCV vs. time profiles is due to the necessarily random formation of pinholes through which reactant gas crosses over, leading to random drops in OCV and eventually, failure. In light of such randomness, the three profiles shown are quite similar.

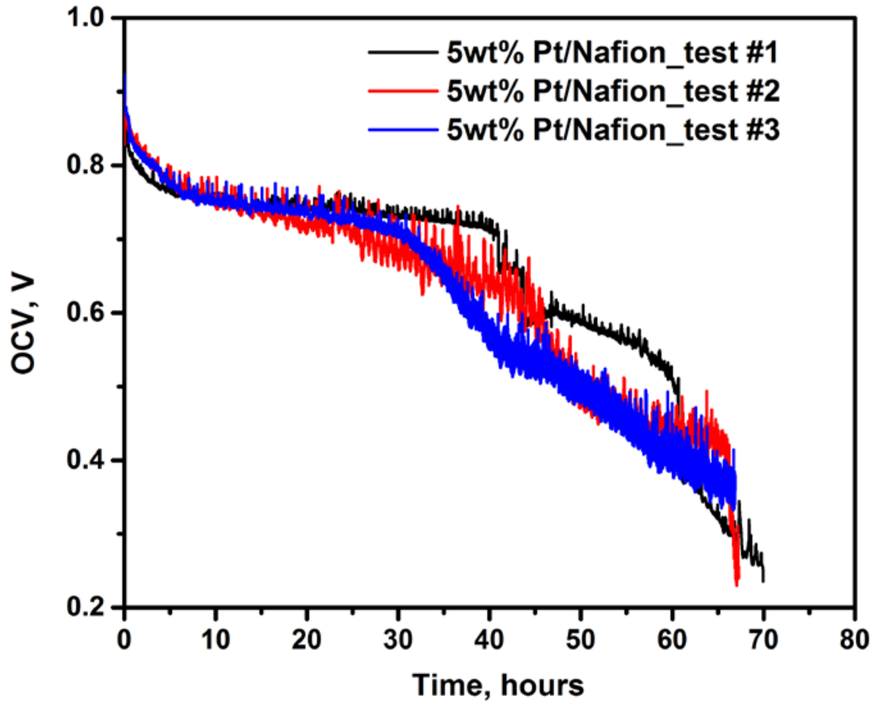

Supplementary Figure 11. Accelerated fuel cell durability tests of the Nafion composite membranes with 5wt.% Pt NPs.

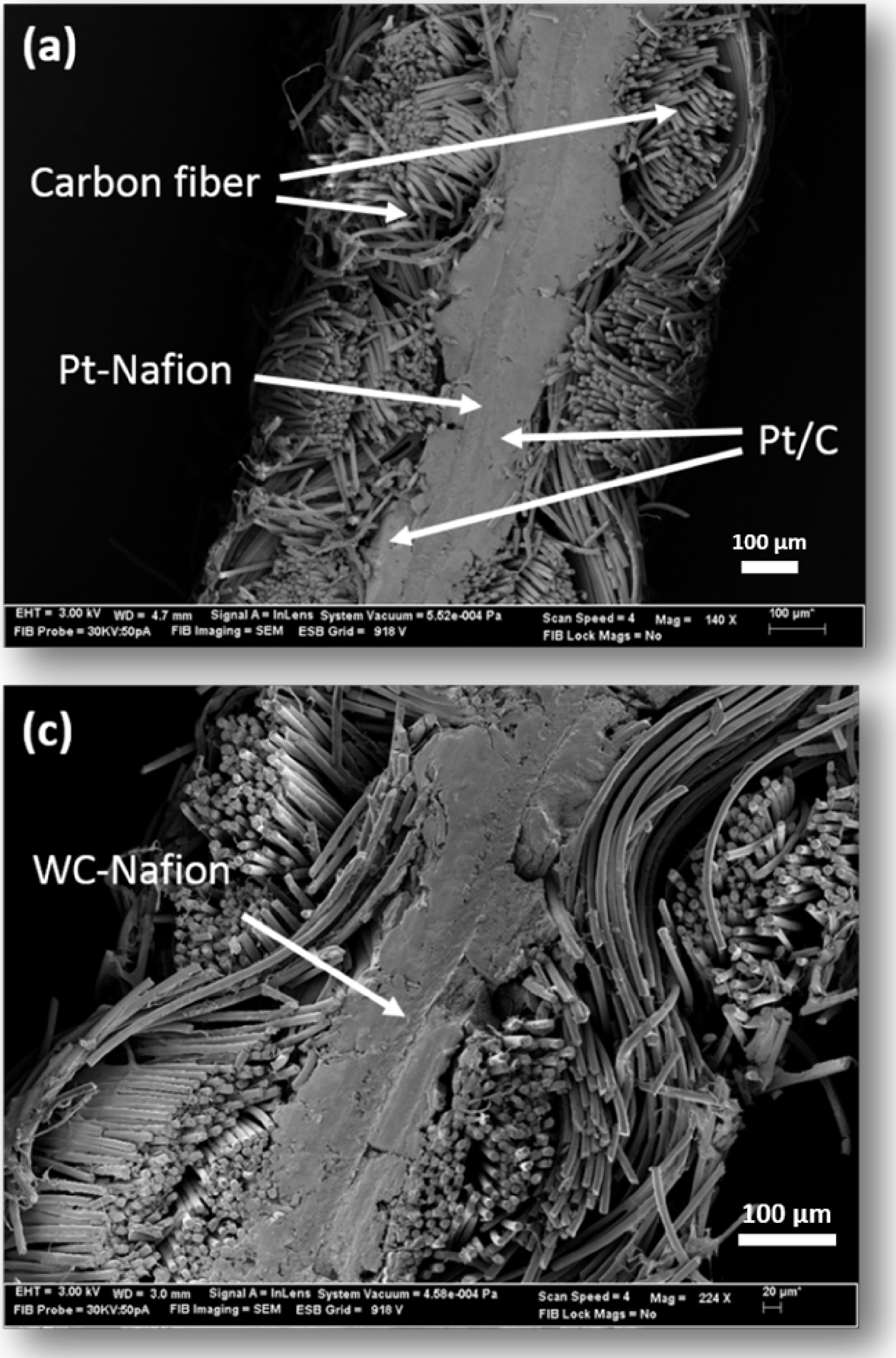

Supplementary Figure 12. Cross-sectional SEM image of Pt/Nafion and WC/Nafion membranes collected after 100 hours of accelerated durability testing.

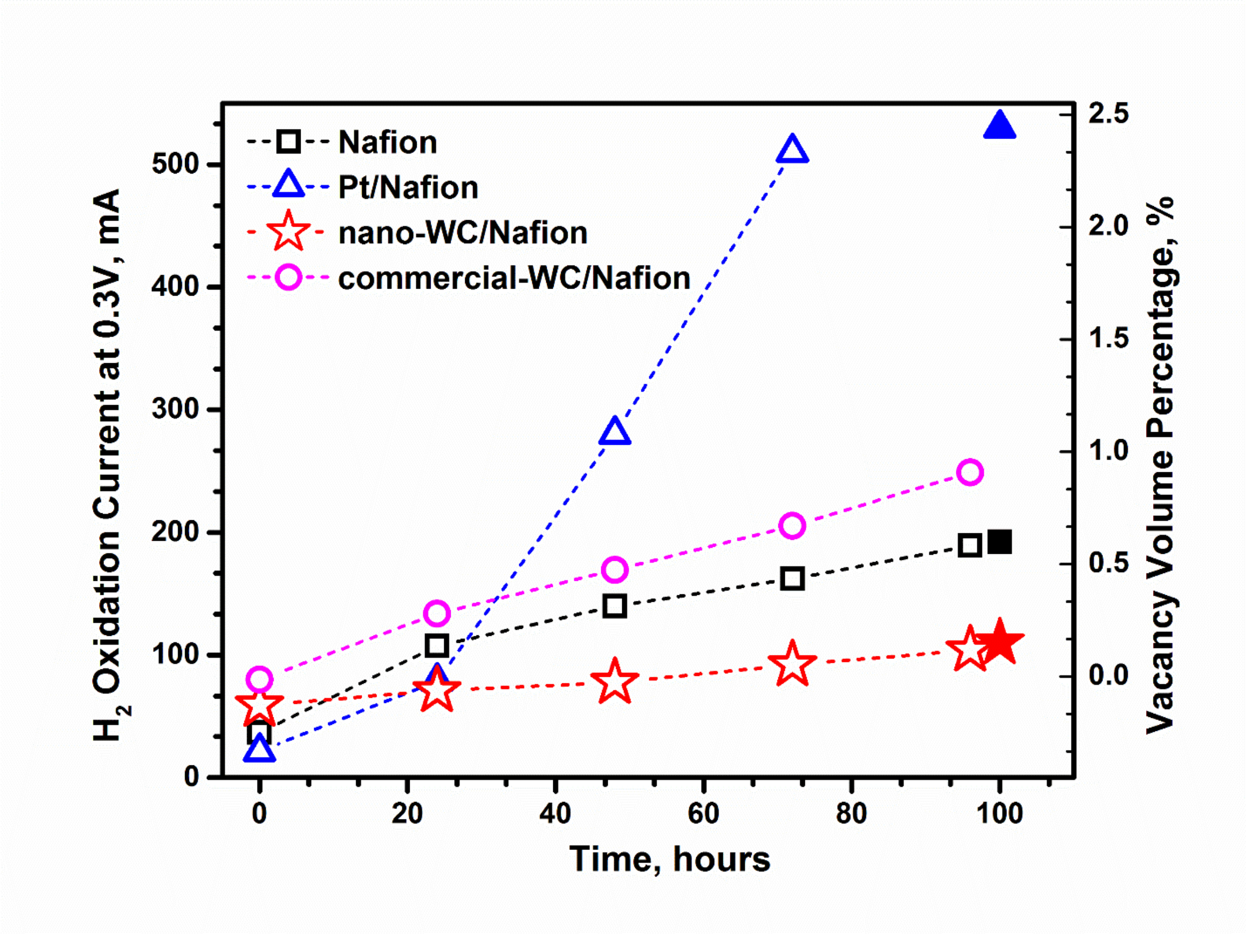

Supplementary Figure 13. Gas crossover of recast Nafion measured (open symbols) and vacancy volume percentages estimated by the tomography of recast Nafion membranes after 100 hours durability tests (closed symbols). Gas crossover was tested by the linear scan voltammetry (LSV) method from 0-0.7V with Scan Rate of 2 mV/s on an AMETEK Versa STAT 3 station using 100% RH nitrogen and hydrogen in the working and counter electrodes, respectively. The hydrogen electrode was also used as the reference electrode. Nitrogen and hydrogen flow rates were both set to 100 mL/min. The H_2 crossover from reference electrode surface to working electrode surface was then oxidized when H_2 moves away from the surface and new H_2 molecules come into contact with the surface of the working electrode. H_2 oxidation current at 0.3 V was used to compare the H_2 crossover of different membranes. Pt/Nafion showed the fastest increase of gas crossover during the durability test due to fastest degradation of the membrane. The nano-WC/nafion membrane is the most stable one with the least gas crossover.

Due to the large scale of composite membranes and strong beam damage to the Nafion structure, we have implemented a Focused Ion Beam – Scanning Electron Microscope (FIB-SEM) to visualize the membrane degradation after 100 hours of accelerated durability tests. The pinhole or

vacancy, Nafion and catalyst can be distinguished by the differences in their contrast in each slice of SEM images. After alignment of all sequential images, we reconstruct the rendering surfaces of vacancy and catalyst of the composite membrane (**Figure 4**). We also measure the volume of the reconstructed pinhole features; see **Supplementary Figure 13** (close symbols).

Supplementary Note 3. First-principles calculations

Determination of lowest energy surfaces. The (111) and (100) surface of Pt and WC, respectively, were chosen as model surfaces due to having the lowest surface energy among the low-index surfaces. Pt (111) is widely reported to have the lowest surface energy of the Pt surfaces. The energy of the (111), (100), and (110) surfaces of WC was computed in accordance with the literature [13]

$$\gamma = \frac{E_{\mathrm{surf}} - N_{\mathrm{bulk}} E_{\mathrm{bulk}}}{2A}$$

where E_{surf} is the total energy from DFT, N_{bulk} is the number of bulk units, E_{bulk} is the energy of one bulk unit, and A is the area of the surface.

The surface energy of each low-index surface of WC is provided in **Supplementary Table 3**.

Supplementary Table 3. Surface energies of the low-index WC surfaces.

Surface	Surface Energy (J m^{-2})
(100)	3.89
(111)	4.03
(110)	4.37

Construction of potential free energy diagrams. The potential free energy diagrams for the *in-situ* production of OH• were constructed following the mechanism of Yu *et al.* Zero-point and entropic corrections to the DFT energies were taken from previously published results.[14] The results obtained for the production of OH• on Pt (111) and WC (100) are provided in **Supplementary Figure 14**. For Pt (111), shown in **Supplementary Figure 14a**, the production of OH• through an adsorbed HOOH* intermediate is mildly uphill in free energy (+0.56 eV). Note that all reaction energies computed herein correspond to the low coverage of 1/9 ML. This thermodynamic barrier could likely be overcome on Pt at higher coverages due to repulsive lateral interactions between adsorbates.

In contrast, the potential free energy diagram for WC (100) (**Supplementary Figure 14b**) indicates that the lowest energy pathway to produce OH• is a co-adsorbed H* and OOH* intermediate whereby the reaction is *strongly endergonic* (+4.01 eV). While the thermodynamic barrier for desorption of OH• will decrease at high coverage due to lateral interactions, previous results indicate that these interaction energies are relatively mild with a pairwise O-O interaction of 0.16 eV on a Pt(100) surface.[15] Therefore, the production of OH• through the mechanism described by Yu *et al.* should be thermodynamically unfavorable even at high coverages on WC (100).

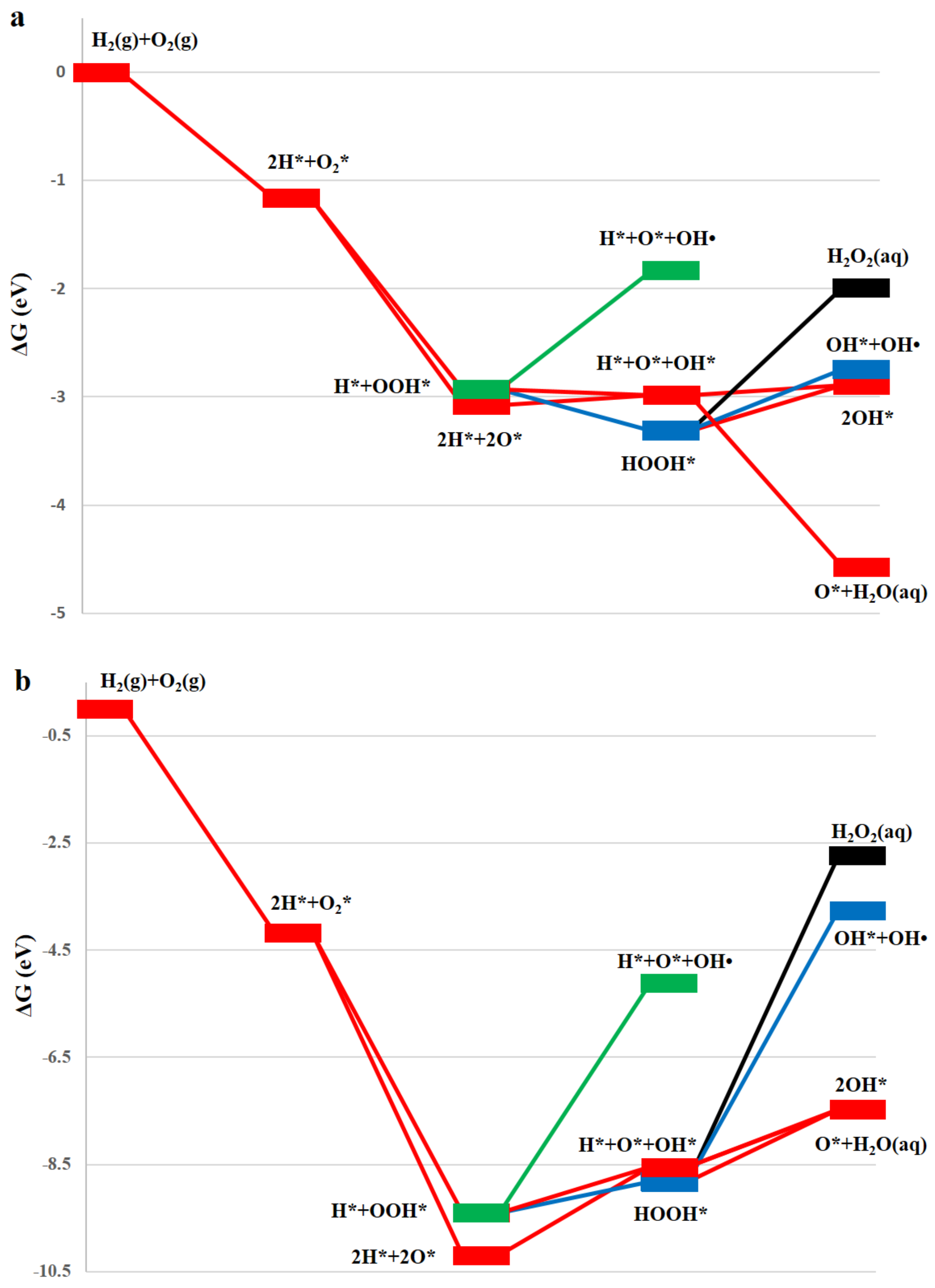

Supplementary Figure 14. Potential free energy diagram for the formation of OH• from H_2 and O_2 on (a) Pt (111) and (b) WC (100).

Supplementary Note 4. Assessment of new materials as electrode catalyst

In order to further assess the reactivity of nano-WC, we have tested our catalyst in a fuel cell as electrodes. The results (**Supplementary Figure 15**) show similar performance using nano-WC catalyst on the anode and cathode. The peak power density of the fuel cells with nano-WC is about 0.09 W/cm^2. The results indicate catalytic activity of nano-WC catalyst for H_2 oxidation and O_2 reduction.

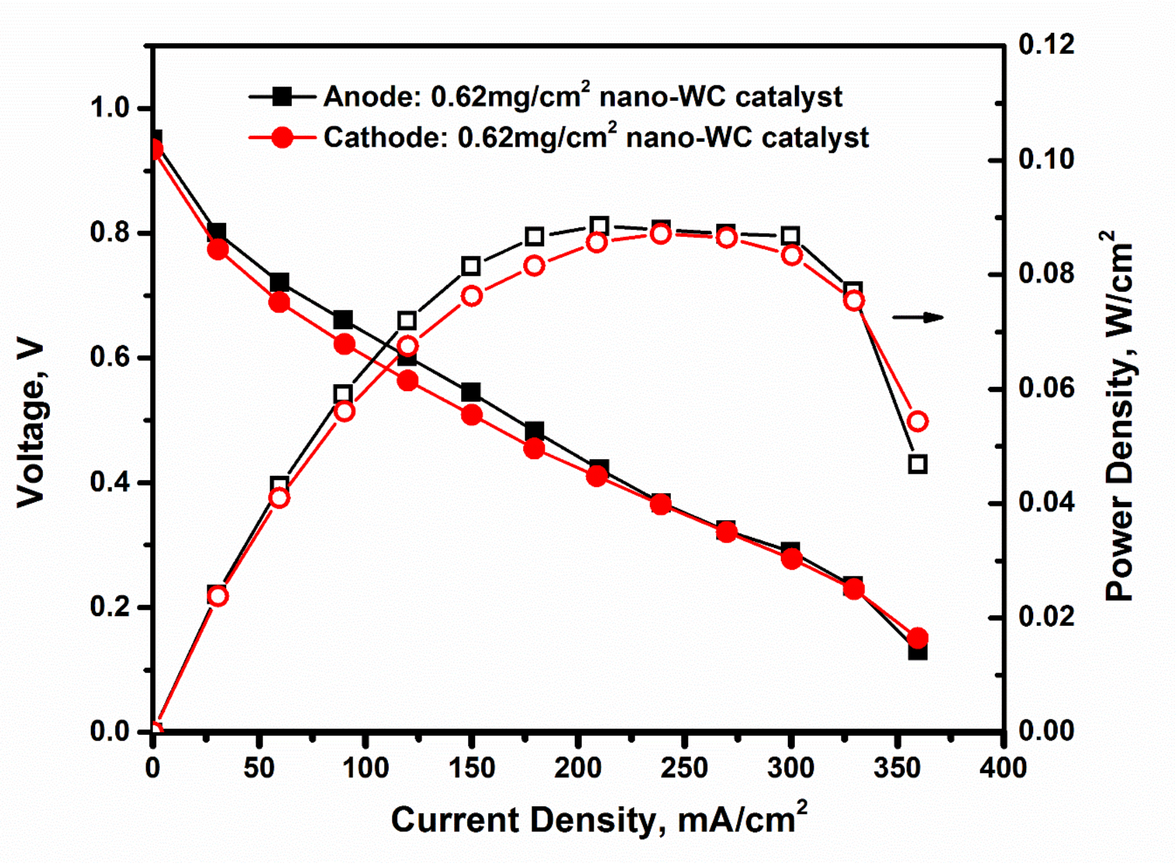

Supplementary Figure 15. Polarization curves of fuel cells using nano-WC catalyst as anode catalyst (black) and cathode catalyst (red). The electrode with nano-WC catalyst was prepared by air spraying nano-WC, Nafion and isopropyl Alcohol (IPA) mixture onto commercial gas diffusion media (carbon cloth with microporous layer). The loading of nano-WC is 0.62 mg/cm^2 and the loading of Nafion is 25 wt%. The fuel cells were tested with (1) home-made nano-WC electrode on the anode and commercial Pt/C electrode (0.3 mg/cm^2) on the cathode and (2) nano-WC electrode on the cathode and commercial Pt/C electrode (0.3 mg/cm^2) on the anode. The testing temperature of the cell is 70 °C, 100% RH with 200 ml/min H_2 and 400 ml/min O_2.

References

1 Lee, J. S., Oyama, S. T. & Boudart, M. Molybdenum carbide catalysts : I. Synthesis of unsupported powders. *J. Catal.* **106**, 125-133, (1987).

2 Berl, E. & Schmidt, A. Über die Entstehung der Kohlen. II. Die Inkohlung von Cellulose und Lignin in neutralem Medium. *Justus Liebigs Annalen der Chemie* **493**, 97-123, (1932).

3 Titirici, M.-M., Antonietti, M. & Baccile, N. Hydrothermal carbon from biomass: a comparison of the local structure from poly- to monosaccharides and pentoses/hexoses. *Green Chem.* **10**, 1204-1212 (2008).

4 Titirici, M.-M. & Antonietti, M. Chemistry and materials options of sustainable carbon materials made by hydrothermal carbonization. *Chem Soc Reviews* **39**, 103, (2010).

5 Cui, X., Antonietti, M. & Yu, S.-H. Structural Effects of Iron Oxide Nanoparticles and Iron Ions on the Hydrothermal Carbonization of Starch and Rice Carbohydrates. *Small* **2**, 756-759, (2006).

6 Sun, X. & Li, Y. Colloidal Carbon Spheres and Their Core/Shell Structures with Noble-Metal Nanoparticles. *Angew. Chem. Int. Ed.* **43**, 597-601, (2004).

7 Krasovskii, P. V. *et al.* XPS study of surface chemistry of tungsten carbides nanopowders produced through DC thermal plasma/hydrogen annealing process. *Appl. Surf. Science* **339**, 46-54, (2015).

8 Ganesan, R. & Lee, J. S. Tungsten carbide microspheres as a noble-metal-economic electrocatalyst for methanol oxidation. *Angew Chem Int Ed Engl* **44**, 6557-6560, (2005).

9 Yan, Z., Cai, M. & Shen, P. K. Nanosized tungsten carbide synthesized by a novel route at low temperature for high performance electrocatalysis. *Scientific Reports* **3**, 1646, (2013).

10 Oyama, S. T. in *Handbook of Heterogeneous Catalysis* (Wiley-VCH Verlag GmbH & Co. KGaA, 2008).

11 Watanabe, M., Uchida, H., Seki, Y., Emori, M. & Stonehart, P. Self‐Humidifying Polymer Electrolyte Membranes for Fuel Cells. *J. Electrochem. Soc.* **143**, 3847-3852 (1996).

12 U.S. Department of Energy, *Cell Component Accelerated Stress Test Protocols for PEM Fuel Cells*, http://www1.eere.energy.gov/hydrogenandfuelcells/fuelcells/pdfs/component_durability_profile.pdf (2007).

13 Seriani, N., Pompe, W. & Ciacchi, L. C. Catalytic Oxidation Activity of Pt3O4 Surfaces and Thin Films. *J. Phys. Chem. B* **110**, 14860-14869, (2006).

14 Man, I. C. *et al.* Universality in Oxygen Evolution Electrocatalysis on Oxide Surfaces. *ChemCatChem* **3**, 1159-1165, (2011).

15 Ge, Q. *et al.* Site symmetry dependence of repulsive interactions between chemisorbed oxygen atoms on Pt{100}-(1×1). *J. Chem. Phys.* **106**, 1210-1215, (1997).
